# *C. auris* in Wastewater: Current Evidence, Risks, One Health Implications, and Knowledge Gaps

**DOI:** 10.1021/acsomega.6c02347

**Published:** 2026-06-15

**Authors:** Alcino Trindade Rosa-Machado, Bruna Coelho Lopes, César Mota Filho

**Affiliations:** Department of Sanitary and Environmental Engineering, Federal University of Minas Gerais, 6627 Avenida Antônio Carlos, Pampulha, Belo Horizonte, Minas Gerais 31270-901, Brazil

## Abstract

*Candidozyma aurisauris* (formerly *Candida auris*) is an emerging, multidrug-resistant fungal pathogen that is difficult to identify and has become an increasing challenge for global public health. In recent years, its detection in wastewater has raised concerns regarding the potential environmental dimensions of its dissemination and the associated public health implications. This study examines the current evidence on the occurrence of *C. auris* in wastewater, with an emphasis on the concentration, isolation, and identification methodologies employed in recent investigations. Framed within a One Health perspective, the analysis discusses potential pathways of environmental dissemination through wastewater effluents and biosolids, particularly in the context of the expanding reuse of treated wastewater and the land application of sewage sludge. The review also highlights existing regulatory gaps, including the absence of specific guidelines addressing pathogenic fungi in wastewater treatment plant byproducts as well as the lack of standardization in reported data, which hinders more in-depth analyses. Overall, this work identifies important knowledge gaps and emphasizes the need for further studies and interdisciplinary surveillance strategies to better understand the environmental circulation of *C. auris*. Additionally, a conceptual workflow is proposed to advance the standardization of analytical approaches and data reporting, contributing to strengthening public health, environmental protection, and sanitary policies.

## Graphical abstract

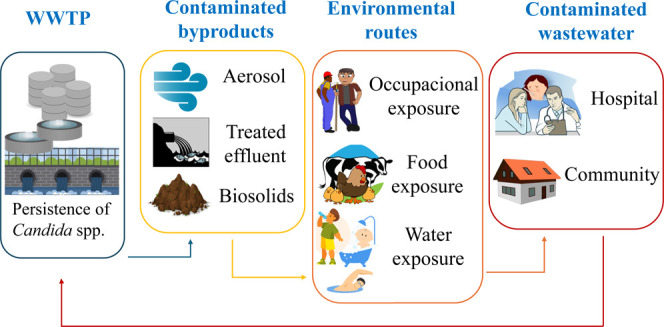

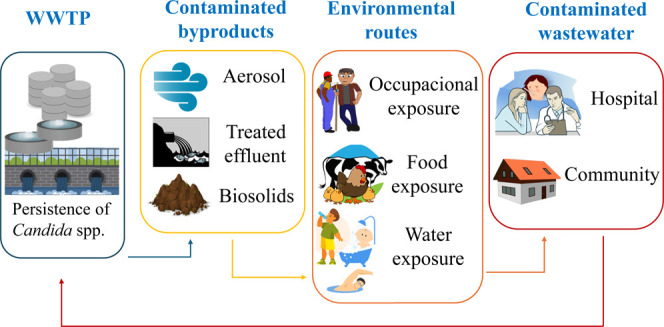

## 1 Introduction

Infectious diseases remain one of the leading causes of disability and mortality worldwide, imposing major burdens on health care systems, food production, and the environment. Global antimicrobial resistance (AMR) initiatives have primarily focused on bacterial pathogens, overlooking fungi despite their expanding clinical and environmental relevance. In this context, invasive fungal diseases (IFDs) are now recognized as an escalating global crisis, particularly among immunocompromised and critically ill patients.^1^ More than 300 million individuals are affected by serious fungal infections, and over 1.5 million die annually—figures comparable to tuberculosis or malaria.^2^

Despite increasing awareness, fundamental knowledge gaps persist regarding the global burden, resistance dynamics, and environmental dissemination of fungal pathogens.^3^ The WHO Fungal Priority Pathogens List (FPPL) explicitly highlights the scarcity of high-quality data on fungal disease distribution and antifungal resistance, largely due to limited diagnostic capacity and the absence of standardized surveillance systems, particularly in low- and middle-income countries.^4^ *Candidozyma auris* (formerly *Candida auris*) is an emerging, multidrug-resistant fungal pathogen that is difficult to identify and has become an increasing challenge for global public health. In recent years, its detection in wastewater has expanded concerns regarding the environmental dimensions of its dissemination and associated public health risks.

This review explores why *C. auris* remains one of the “missing links” in One Health surveillance frameworks and how its emergence challenges the traditional separation between clinical and environmental domains. By examining current evidence on its ecological niches, resistance mechanisms, and persistence in hospital and wastewater environments, we provide a critical review based on an integrated One Health perspective that connects human, environmental, and infrastructural drivers of fungal dissemination.

## 2 Ecological Niches and Adaptive Traits of *C. auris*

Among the fungal pathogens listed as critical priorities by the World Health Organization,^4^ *C. auris* stands out as an unprecedented global health threat. Since its first identification in 2009, this opportunistic yeast has been responsible for numerous hospital outbreaks across all inhabited continents, demonstrating a capacity for nosocomial persistence, antifungal resistance, and intercontinental dissemination.^1,5^ Initially grouped into five major phylogeographic clades—South Asian (Clade I), East Asian (Clade II), African (Clade III), South American (Clade IV), and Iranian (Clade V)—the species’ diversity has recently expanded with the discovery of a sixth distinct clade (Clade VI) identified in Singapore and Bangladesh.^6^

Beyond this genetic diversity, *C. auris* exhibits remarkable ecological versatility and physiological resilience that underpin its emergence as both a clinical and environmental organism. Similar to *C. albicans*, *C. auris* also has several cellular morphologies, including yeast-like, elongated, and filamentous forms.^3,7^ Morphological transitions appear to be a conserved feature among clinical isolates belonging to distinct genetic clades, suggesting that phenotypic plasticity is an inherent adaptive trait of this species.^7^

Such morphological transitions are thought to enhance environmental persistence, promote host tissue colonization, and facilitate evasion of immune responses. In conjunction with its broad-spectrum antifungal resistance, this dynamic capacity for morphological switching likely contributes to the remarkable adaptability, global spread, and clinical success of *C. auris*.^7^ Complementing these traits, experimental studies have shown that *C. auris* thrives across a wide temperature range of 25–42 °C^3,7^ and tolerates salinity levels above 10% NaCl, conditions uncommon among *Candida* species.^3^ Such thermotolerant and halotolerant characteristics further reinforce the notion that *C. auris* is ecologically preadapted to survive both in clinical environments and in external settings subject to fluctuating physicochemical stressors.

The expanding ecological footprint of *C. auris* across humans, animals, and environmental reservoirs supports its potential relevance within a One Health framework. Traditionally confined to healthcare facilities, *C. auris* has now been isolated from multiple nonhuman hosts and environmental matrices, indicating its capacity to adapt to phylogenetically diverse organisms and persist under environmental stress.

In the Dominican Republic, *C. auris* was simultaneously detected in a critically ill human and a captive bottlenose dolphin (Tursiops truncatus), both carrying Clade I strains with high genomic similarity—constituting the first evidence of cross-species occurrence within the same geographic region.^8^ In Europe, a persistent intestinal colonization was reported in a domestic cat (*Felis catus*) from Italy, shedding viable *C. auris* cells in feces over six months without clinical disease or antifungal exposure.^9^

Recent findings further expand this host range to companion animals, particularly dogs. In India, *C. auris* was isolated from the ear and skin of dogs with chronic otitis externa and dermatological lesions, with whole-genome sequencing confirming Clade I strains genetically related to regional human isolates.^10^ Similarly, in the United States, *C. auris* was detected for the first time in a nonhuman mammal—a domestic dog from Kansas—colonizing the oral cavity. The isolate belonged to Clade IV, displayed multidrug resistance to fluconazole, amphotericin B, and terbinafine, and represented the first detection of *C. auris* in an animal host in North America.^11^

These findings collectively demonstrate that *C. auris* can colonize both marine and terrestrial mammals, suggesting potential zoonotic interfaces shaped by anthropogenic and environmental pressures. Parallel environmental detections have revealed *C. auris* DNA in wastewater, hospital effluents, and marine sediments.^12^

To summarize the current evidence supporting this One Health continuum, Table 1 compiles documented reports of *C. auris* across human, animal, and environmental matrices worldwide, including both culture-based isolation and molecular detection by quantitative polymerase chain reaction (qPCR). This comparative overview illustrates how genetically related lineages of *C. auris* have been recovered from hospitals, domestic and marine hosts, and diverse ecological compartments, including untreated sewage, treated effluents, and coastal sediments, demonstrating that *C. auris* has evolved into a transdomain pathogen capable of bridging clinical and environmental ecosystems.

**Table 1:** Tracing the One Health Distribution of *Candidozyma auris* across Clinical and Environmental Reservoirs

Domain	Host/Source/Matrix	Location	Key Findings	References
Human (clinical)	External ear canal of a hospitalized patient	Japan	First isolation reported on human	^13^
Human (clinical)	Intensive care unit patient with bloodstream infection	Dominican Republic	Fluconazole resistant (>64 μg/mL)	^8^
Animal (marine)	Dolphin (*Tursiops truncatus*). Pharyngeal specimen	Dominican Republic	Clade I. Rare identification in a marine mammal	^8^
Animal (companion)	Domestic cat (*Felis catus*)	Italy	Persistent fecal shedding (6 months); multidrug resistant (Fluconazole, Amphotericin B, Caspofungin)	^9^
Animal (companion)	Dog (*Canis familiariz*)—ear and skin swabs	India	Clade I. Chronic skin disease with otitis externa	^10^
Animal (companion)	Dog (*Canis familiariz*)—oral cavity	USA	Clade IV. First detection in North America; multidrug resistant (fluconazole, terbinafine, and amphotericin B)	^11^
Environmental (Seawater)	Salt marsh and sandy beach	India	Clade I. multidrug-susceptible and multidrug-resistant	^14^
Environmental (WWTP)	Wastewater	USA	*C. auris* DNA detected by qPCR	^12^
Environmental (WWTP)	Wastewater	USA	Prospective study of *C. auris* DNA detected by qPCR in 190 WWTP	^15^

Taken together, the available evidence remains limited but suggests the possibility of a complex transmission network linking environment → animal → human. However, this hypothesis should be interpreted with caution, as current data are insufficient to consistently support this dynamic. In this context, integrated surveillance approaches, including those based on wastewater treatment plants (WWTPs), may represent a promising strategy.

## 3 Knowledge Gaps and Research Needs

Despite more than a decade of research since *C. auris* was first isolated in Japan,^13^ key aspects of its ecology, transmission, and resistance evolution remain poorly understood. Addressing these gaps is essential for developing an integrated One Health framework that connects clinical, veterinary, and environmental surveillance.

### 3.1 Environmental Reservoirs and Persistence in Natural and Engineered Systems

*C. auris* has traditionally been regarded as a hospital-associated pathogen;^8,13,16^ however, emerging evidence suggests its ability to persist beyond clinical settings. The detection of *C. auris* DNA in diverse environmental matrices (Table 1) points to the existence of potential environmental reservoirs capable of sustaining viable yeast cells or preserving genetic material for extended periods. This limited understanding of the environmental ecology and life cycle of *C. auris* constrains our ability to explain its abrupt global emergence and continued spread.^17^ Further research is, therefore, required to elucidate the mechanisms that enable this yeast to withstand the physicochemical conditions of environmental matrices and to assess the implications for environmental transmission and public health.^17^

Unlike *C. albicans* and most other *Candida* species, which are mainly commensal microbes in the gut, vagina, and other internal niches, *C. auris* is unable to survive or persist under anaerobic conditions.^18^ Nonetheless, its detection in marine and coastal environments provides evidence that *C. auris* can also persist outside clinical settings, suggesting the existence of environmental reservoirs that may contribute to its dissemination.^14^

*C. auris* exhibits tolerance to abiotic stress, including growth at elevated temperatures (up to 42 °C), which may support its persistence in environmental systems.^14,19^ In addition, a study shows that *C. auris* can resist oxidative stress induced by hydrogen peroxide (H_2_O_2_) compared to *C. albicans* and *C. dubliniensis*, whereas *C. glabrata* displays the highest overall tolerance to H_2_O_2_ but is especially resistant to cationic stress imposed by sodium and calcium chloride.^18^ This physiological behavior has important implications for wastewater treatment systems, where oxidative agents such as H_2_O_2_ are widely used in disinfection and advanced oxidation processes (AOPs) to remove surfactants and emerging contaminants.^20^ Given *C. auris* demonstrated resistance to oxidative damage, residual H_2_O_2_ from AOPs could exert selective pressure favoring its survival in post-treatment environments, suggesting that WWTPs might function not only as treatment barriers but also as ecological niches supporting stress-tolerant fungal species.^18,20^

These physiological characteristics may allow the yeast to survive in wastewater treatment systems operating at the quaternary level, where temperature fluctuations, high salinity, and residual oxidants, such as hydrogen peroxide, are common. Within wastewater treatment processes, such selective pressures could contribute to the maintenance of stress-tolerant fungal populations and favor their adaptation to post-treatment environments. Considering these factors, wastewater treatment plants may represent potential ecological interfaces between anthropogenic and natural systems, where *C. auris* could persist and be disseminated. This hypothesis provides the basis for the subsequent discussion of wastewater treatment plants as critical nodes in the environmental dissemination network of *C. auris*.

Most estimates of invasive fungal diseases are derived from hospital-based studies with limited integration of environmental surveillance. At the environmental level, systematic monitoring of resistant fungi remains neglected in global antimicrobial resistance frameworks, despite mounting evidence suggesting that ecological reservoirs, including soil, surface waters, and wastewater, may serve as critical sources and sinks for antifungal-resistant strains. This lack of integration between clinical and environmental surveillance prevents a comprehensive understanding of fungal transmission pathways and hinders the application of One Health principles to fungal diseases.^1,21^

*C. auris* exhibits traits such as thermotolerance and antifungal resistance, which may contribute to its persistence beyond clinical environments.^12,22^ Consequently, the real contribution of environmental dissemination to the persistence and reemergence of pathogens, such as *C. auris*, remains one of the most urgent and unresolved challenges in infectious disease research.

### 3.2 Wastewater Treatment Plants (WWTPs) as Critical Nodes

Antimicrobial resistance (AMR) represents a major public health challenge with far-reaching consequences that extend beyond human health, encompassing increased healthcare costs as well as impacts on veterinary medicine, agriculture, and the environment.^23^ Despite its global relevance, efforts to monitor and understand AMR remain constrained by substantial gaps in data collection, surveillance, and research capacity.^24,25^ In response to these limitations, the World Health Organization established the Global Antimicrobial Resistance Surveillance System (GLASS) to systematically collect, analyze, and share AMR data worldwide.^26,27^ GLASS operates under the One Health framework, which recognizes the interconnectedness of human, animal, and environmental health.^4,28^ However, the operationalization of One Health in AMR surveillance has largely remained centered on human health, with persistent gaps in the integration of environmental compartments, animal reservoirs, and the role of wastewater treatment plants (WWTPs) in the emergence and dissemination of resistance.^29^

Wastewater has been suggested as a potential vector of increased selection and transmission of AMR. As mentioned earlier, because WWTPs are frequently not designed for pathogen removal, these facilities are considered hotspots for the release of antibiotic-resistant bacteria and genes into the environment^30^ and may also contribute to the release of *C. auris* and other pathogenic fungi into the environment.

Although studies have focused on WWTPs as elements for surveillance and management of antibiotic resistance under the One Health perspective,^31^ these studies have focused on the context of bacterial antimicrobial resistance. In addition to their sanitary function, WWTPs can also play a strategic role in the environmental surveillance of the spread of resistant pathogens, especially fungal ones, whose dynamics are still little explored through the lens of the One Health approach. The impacts on the health of vulnerable populations living near the receiving bodies of WWTPs and using water from these sources for consumption and daily activities need to be clarified. Moreover, an aggravating factor is that in regions lacking efficient wastewater collection and transportation systems, these rivers receive untreated sewage discharges, which represent even higher microbiological risks.

Furthermore, increasing water scarcity in many regions of the world has intensified the reliance on wastewater reuse, raising concerns about the potential dissemination of pathogenic fungi present in treated effluents. In parallel, the reuse of wastewater treatment plant byproducts, such as digested sludge, has been promoted as a circular economy strategy aimed at resource recovery. However, these practices must be implemented with careful consideration of environmental safety and public health protection. Several fungal genera that include species pathogenic to humans and plants—such as *Olpidium*, *Paecilomyces*, *Aspergillus*, *Rhodotorula*, *Penicillium*, *Candida*, *Synchytrium*, *Phyllosticta*, and *Mucor*—have been reported in WWTP influent, highlighting the potential for wastewater systems to act as reservoirs and dissemination pathways for fungal pathogens.^32^

Moreover, the presence of pathogenic fungi classified as high priority by the World Health Organization, such as *Aspergillus* spp. and *Candida* spp., has already been reported in water samples from a coastal ecosystem in southern Brazil. During 25 months of monitoring, *Aspergillus* from the *Fumigati* section and *Candida parapsilosis* were the most frequently isolated species, with resistance to triazoles observed in some of these isolates, reinforcing the role of coastal aquatic environments as potential reservoirs and sources of infection by resistant fungi.^33^ Additionally, the presence of *Aspergillus* spp. and *Candida* spp. has also been reported in the sands of coastal beaches and freshwater recreational areas.^34^

The occurrence of these pathogens in these ecosystems may be strongly associated with the discharge of sewage and excreta into watersheds that drain into coastal areas. Rivers that pass through urbanized areas often receive effluents from both WWTPs and irregular untreated discharges, especially in regions with poor sewage infrastructure. This scenario favors the introduction and dispersion of resistant microorganisms such as pathogenic yeasts in coastal ecosystems. Studies that evaluated resistomes upstream and downstream of rivers exposed to WWTP effluents have revealed that effluents certainly affect the diversity and abundance of the resistome.^35–37^

Moreover, in coastal cities, it is common to use submarine outfalls for the final disposal of partially treated sewage, which may increase the resistant microbial load released directly into the marine environment. Therefore, understanding the contribution of WWTPs and sanitation systems as indirect vectors for the spread of pathogenic fungi in coastal environments is crucial for strengthening environmental surveillance strategies from a One Health perspective.

The presence of *Candida* spp. in aquatic environments also raises concerns about the potential contamination of fish, as *C. albicans* has been reported in aquaculture systems.^38^ Fungal infections in fish are generally considered secondary to other factors or pathogens, often due to water quality issues.^39^ Furthermore, resistant bacteria have been isolated in aquaculture systems.^40^ The presence of pathogenic fungi and the potential emergence of fungal resistance in aquaculture draw attention to the contamination of fish in rivers that receive treated sewage from WWTPs, as it may be a source of infections through the food chain, representing a public health concern. However, the impacts of WWTPs on the contamination of aquatic fauna by fungal diseases due to the discharge of treated sewage still require further studies.

The presence of *C. auris* in sewage also raises important concerns regarding the occupational health for workers at WWTPs. Without the proper use of personal protective equipment (PPE), WWTP operators may be chronically exposed to bioaerosols or materials contaminated with viable yeasts, especially during treatment stages involving direct contact with raw sewage or sludge or during equipment cleaning. Viable fungi such as *Clostridium perfringens* and *Aspergillus fumigatus* have been found in aerosol samples collected in WWTPs.^41^ Furthermore, fungi from the genera *Cochliobolus* spp., *Sclerotinia*, and *Aspergillus* have been identified in aerosols formed during the composting of vegetable waste.^42^ One of the methods used for stabilizing sludge produced in WWTPs before its application to the soil is composting. However, no studies have yet addressed the release of pathogenic fungi, including *Candida* spp., from WWTP sludge composting units. The absence of appropriate PPE, insufficient ventilation, and negligence in the application of strict sanitary protocols in WWTPs can increase the risk of fungal colonization and infection. Thus, it is important to incorporate fungal occupational risk assessments into biosafety protocols and sanitary surveillance programs in WWTPs, aligning with the One Health principles, which consider the interconnection among human, animal, and environmental health.

In the context of Brazilian legislation, the National Environment Council (CONAMA) Resolution n°. 430/2011 establishes water quality standards for effluent discharge into water bodies, including specific limits for total coliforms.^43^ However, the regulation does not address parameters related to the presence of fungi in treated effluents, which represents a significant gap, given the increasing concern about pathogenic fungi and fungal resistance that may be released into the environment through WWTPs.

Although the agricultural reuse of treated wastewater is widely promoted as a strategy aligned with circular economy principles and sustainable water resource management, the current regulations present significant gaps. Both the European Union Regulation (EU) 2020/741 and the U.S. guidelines for water reuse (EPA/600/R-12/618), which establish minimum microbiological quality requirements for wastewater reuse, do not include parameters for the detection of pathogenic fungi, such as those belonging to the *Candida* spp. genus, nor do they address aspects related to fungal resistance in treated effluents.^44,45^ This regulatory gap is concerning, given the increasing emergence of opportunistic fungi, such as *C. auris*, which are recognized for their potential for environmental dissemination and impact on public health.

Studies show that soil is a reservoir for antibiotic resistance genes.^46,47^ The presence of *C. auris* in biosolids is also not regulated for soil application purposes.^48–50^ In Brazil, the Resolution CONAMA n°. 498/2020 and IN 07/2016 establish criteria for the agricultural use of sewage sludge, regulating specific microbiological parameters such as thermotolerant coliforms, viable helminth eggs, and *Salmonella* spp.^51,52^ However, the regulation limits its fungal target to phytopathogenic species from the genera *Fusarium*, *Phytophthora*, *Pythium*, *Rhizoctonia*, and *Sclerotinia*, completely ignoring the presence and potential risk of emerging opportunistic fungi, such as *C. auris*. The application of sludge to agricultural soils and the use of treated effluent for irrigation, for example, can favor the persistence of viable yeasts in the environment, enabling their entry into ecological and food chains. Although there is no documented evidence of the presence of *C. auris* in WWTP sludges or composts, the lack of data should not be interpreted as the absence of risk.

In this sense, the consideration of opportunistic fungi such as *C. auris* is recommended in regulatory and scientific agendas, in both Brazilian and international guidelines for agricultural reuse and environmental disposal of effluents.

### 3.3 Contribution of WWTPs to the Control of *C. auris* in the Environment and Current Challenges

Wastewater treatment plants can play a fundamental role in reducing the spread of pathogens in the environment. However, current knowledge regarding the presence of *C. auris* in domestic influent samples entering WWTPs remains limited,^12^ as well as its occurrence in treated effluent and its partitioning throughout the treatment process. To the best of our knowledge, only one study to date has reported the removal of *C. auris* in WWTPs, with a removal efficiency of approximately 1.24 log_10_ in primary sedimentation units,^12^ and no studies have evaluated its removal in secondary treatment units. Given these limitations, the potential persistence of *C. auris* in treated effluents and its implications for environmental and public health remain uncertain and should be interpreted with caution. In this context, potential impacts may include exposure of populations in direct contact with receiving water bodies affected by wastewater discharges, such as riparian communities, although such risks have not yet been well established in the literature. In addition, while many wastewater treatment systems, particularly in low- and middle-income countries, are primarily designed to remove organic matter and solids, their effectiveness in removing emerging fungal pathogens such as *C. auris* has not yet been established.

Among the various pathogen removal mechanisms occurring in WWTPs, one potential pathway for *C. auris* removal is adsorption onto settleable solids, as indicated by the removals reported in primary sedimentation.^12^ The interaction between dense microbial communities and settled sludge, which is common in biological wastewater treatment reactors, may provide favorable conditions for the horizontal transfer of resistance genes.^53^ Furthermore, in activated sludge systems, when sludge is recirculated to the aeration tank, resistance genes may be reintroduced into the system, perpetuating this dissemination cycle.^54^ Therefore, it is necessary to assess the behavior of resistant fungi throughout the treatment process to determine whether wastewater matrices and WWTPs may act as environments for the selection of antifungal resistance.

A study conducted in South Africa investigated the presence of azole-resistant fungi in treated effluents from two WWTPs. Twenty-three fungal species belonging to 16 genera were identified, including clinically and environmentally relevant pathogens such as *Aspergillus* spp., *Fusarium* spp., and *Candida pseudolambica*. Susceptibility testing revealed resistance of *Aspergillus fumigatus* to fluconazole, while *Fusarium oxysporum* showed resistance to three azoles: ketoconazole, itraconazole, and voriconazole. Despite this, voriconazole exhibited the highest antifungal activity against both isolates. *Candida parapsilosis* ATCC 2209 and *Candida krusei* ATCC 6258 strains presented MIC ≤ 1 μg/mL for all antifungals tested, except fluconazole (FCZ), for which the MIC for *C. krusei* was 64 μg/mL. Genetic analysis of the *Cyp51A* gene in *A. fumigatus* did not indicate mutations associated with resistance, suggesting the involvement of other mechanisms.^32^ However, the authors did not specify the treatment technology employed by the WWTPs assessed.

Accordingly, it is essential to promote further studies to understand the removal mechanisms occurring in these systems, as they may support both process optimization and informed decision-making when selecting treatment technologies for implementation.

To achieve proper pathogen removal and inactivation, tertiary treatment units such as chlorination and ultraviolet-C (UV-C) disinfection may be required. Studies have reported removals of approximately 6 log_10_ for *C. auris*, *C. glabrata*, and *C. albicans* after exposure to UV-C light for 30 min at distances of 1.5 and 2.0 m.^55,56^ At a closer distance of 1.0 m, total disinfection has been reported after 15 min of exposure.^57^ The efficiency of UV-C disinfection of *C. auris* is influenced by concentration, exposure time, and lamp proximity.^56^ Organisms from the *Candida* spp. group are larger than bacteria, which may require higher doses to reach the cell nuclei. Additionally, the presence of suspended solids in treated effluents can protect the yeast cell wall, requiring longer UV-C exposure times. To date, the application of UV-C disinfection for fungal pathogens in wastewater, including those belonging to the *Candida* spp. group, has not yet been explored. Therefore, studies are needed to assess different contact times and define optimal dosages as this parameter will directly influence the cost of WWTP implementation and operation.

Advanced oxidation processes (AOPs) have been widely investigated as post-treatment for domestic wastewater, primarily targeting the removal of antimicrobial-resistant bacteria and antimicrobial resistance genes. However, no studies have been identified to date that evaluate the application of AOPs for the removal of fungal pathogens, including resistant yeasts and their respective resistance genes. Thus, it is crucial to develop research that examines the use of AOPs as a polishing stage for biologically treated effluents, with the aim of reducing the environmental dissemination of pathogenic fungi.

### 3.4 Surveillance and Diagnostic Gaps in Wastewater-Based Epidemiology (WBE)

For the detection of *Candida auris* and other yeasts in wastewater samples, different methodological approaches have been applied internationally. Table 2 summarizes the main studies conducted so far, highlighting differences in the concentration, cultivation, and molecular identification methods.

**Table 2:** Studies Found in the Literature Addressing the Presence of *C. auris* in Wastewater

Location	Year	Sample type	Concentration methods	Culture	Positive Samples/Total Samples Collected	Molecular identification and target gene/region	Primers and probes used for qPCR	Concentration log_10_(gc·L^–1^)	References
Southern Nevada, USA	2021–2022	Domestic sewage	Pellet centrifuge	Media salt Sabouraud dulcitol broth and fluconazole 8 ug/mL. HardyCHROM Candida medium at 42 °C.	1/13 *C. auris* colony isolated from the samples (7,7%)	Ilumina NextSeq 2000	N.A.	N.A.	^22^
Southern Nevada, USA	2021–2022	Domestic sewage	Pellet centrifuge	N.A.	72/91 samples (79%)	qPCR–ITS2 region	Forward primer: 5′-CAG​ACG​AAT​CAT​CGA​ATCT-3′;	2.8 to 5.7 log10 gc·L^–1^	^12^
							Reverse primer: 5′-TTT​CGT​GCA​AGC​TGT​AAT​TT3-3′		
							Probe: 5′-FAM-AAT​CTT​CGC​GGT​GGC​GTT​GCA​TTCA-BHQ_1-3′		
190 wastewater treatment plants in the United States	2023–2024	Domestic sewage	Pellet centrifuge	N.A.	65/190 wastewater treatment plants (13.842 samples)	qPCR–ITS2 region	Forward primer: CGC​ACA​TTG​CGC​CTT​GGG​GTA;	N.A	^15^
							Reverse primer: GTA​GTC​CTA​CCT​GAT​TTG​AGG​CGAC;		
							Probe: CTT​CTC​ACC​AAT​CTT​CGC​GGT;		
Miami-Dade County, Florida, USA	2020–2022	Domestic and hospital sewage	Membrane filtration (Pall Corp. #66278, 47 mm diameter, 0.45 μm pore size)	Membrane filtration (Pall Corp. #66278, 47 mm diameter, 0.45 μm pore size) and cultured from the membrane on CHROMAgar medium plates at 42 °C for 48 h. After growth, the colonies were isolated again in CHROMAgar (42 °C for 48 h).	9/19 hospital sewage samples	TaqMan Fast Universal PCR Master Mix (ThermoFisher Sci. Cat# 4352042)—ITS1 and ITS2	Forward primer: CGT​GAT​GTC​TTC​TCA​CCA​ATCT	Hospital sewage samples: 1.3 × 10^5^ gc·L^–1^	^60^
					3/19 wastewater treatment plants		Reverse primer: TAC​CTG​ATT​TGA​GGC​GAC​AAC	WWTP: 1.9 × 10^4^ gc·L^–1^	
							Probe: 5HEX-TTT​GTG​AAT/​ZEN/​GCA​ACG​CCA​CCGC-3IAB​kFQ		
Brazil	2020–2022	Domestic sewage	Pellet centrifuge	Incubated on Sabouraud Dextrose Agar (SDA) medium at 35 °C for 5 days. After CFU quantification, the culture was transferred to Petri dishes containing the chromogenic medium CHROMagar Candida (Difco, Becton-Dickinson and Company, USA). The plates were incubated at 37 °C for 48 h.	The presence of *C. palmioleophila*, *C. albicans*, *C. guilliermondii*, *C. krusei*, *C. tropicalis*, and *C. utiliz* was detected.	Colony PCR targeting the ITS region (primers ITS1 and ITS4)	Forward primer ITS1: CGT​AGG​TGA​ACC​TGC​GG;	N.A.	^58^
						Yeasts were grown on DAS plates at 30 °C for 48 h	Reverse primer ITS4: TCC TCC GCT		
						Isolates were identified by MALDI-TOF MS	TAT TGA TAT GC,		
							Probe: N.A.		
						Sugar assimilation and enzymatic reactions were analyzed using the VITEK 2 system			
Baltimore, Maryland	2022–2023	Domestic sewage	Membrane filtration (Pall Corp. #66278, 47 mm diameter, 0.45 μm pore size)	N.A.	11,81%	qPCR–ITS2 region	Forward primer: 5′-CAG​ACG​AAT​CAT​CGA​ATCT-3′;	1.2 to 7.9 log10 gc·L^–1^	^61^
							Reverse primer: 5′-TTT​CGT​GCA​AGC​TGT​AAT​TT3-3′		
							Probe: 5′-FAM-AAT​CTT​CGC​GGT​GGC​GTT​GCA​TTCA-BHQ_1-3′		
Utah, USA	2022–2023	Domestic sewage	Membrane filtration (Pall Corp. #66278, 47 mm diameter, 0.45 μm pore size)	Salt Sabouraud Dulcitol Broth	28,5%	qPCR–ITS2 region	Forward primer: 5′-CAG​ACG​AAT​CAT​CGA​ATCT-3′;	<2.95 (below LOQ) to 5.2 log_10_ gc·L^–1^	^62^
							Reverse primer: 5′- TTT​CGT​GCA​AGC​TGT​AAT​TT3-3′		
							Probe: 5′-FAM-AAT​CTT​CGC​GGT​GGC​GTT​GCA​TTCA-BHQ_1-3′		
Southern Nevada, USA	2021–2024	Domestic and hospital sewage	Pellet centrifuge	Cultured in YPD medium (Sigma Catalog #Y1375) at 37 °C overnight or in SSDB medium (Thomas Scientific Catalog #CHM01P620) at 42 °C for 48 h. Antifungal susceptibility of *C. auris* was evaluated by determining the Minimum Inhibitory Concentration (MIC)	Hospital wastewater: 95.4% Domestic sewage: 18.4%	qPCR, Isolates were identified by MALDI-TOF MS—Whole-Genome Sequencing	Forward primer ITS2: 5′-CAG​ACG​TGA ATC​ATC​GAA​TCT-3′;	Hospital wastewater: 8.0 log_10_ gc·L^–1^. Domestic sewage: 6.0 log_10_ gc·L^–1^	^59^
							Reverse primer ITS2:5′-TTT CGT​GCA​AGC TGT​AAT TT-3′;		
							Probe: 5′-FAM-AAT CTT CGC GGT GGC GTT​GCA TTC A-BHQ1–3		

The most common concentration methods include pellet centrifugation^12,15,22,58,59^ and membrane filtration.^60–62^ Membrane filtration, using 0.45 μm pore filters, has proven to be effective in retaining fungal cells, while centrifugation has the advantage of being a rapid and easy-to-execute technique for larger sample volumes.

Although studies focused on isolating fungi from influent samples of wastewater treatment plants, it is important to note that general-purpose culture media, such as DAS (Sabouraud dextrose agar), PDA (potato dextrose agar), DRBC (dichloran rose bengal chloramphenicol agar), BIGGY (bismuth glucose glycine yeast agar), and Czapek Dox, have been used. These media, while allowing yeast growth, are not specific for the selective detection or precise differentiation of *Candida* species, especially *Candida auris*. Only two studies used a selective chromogenic medium for *Candida*, such as CHROMagar Candida^58^ and HardyCHROM Candida medium.^22^ The scarcity of studies using specific media for *C. auris* limits the ability to distinguish it from other fungal species that may be present in wastewater. Another gap observed in the literature is the absence of supplementation with antifungals (such as fluconazole and voriconazole) in culture media, which could promote positive selective pressure, favoring the growth of *C. auris* strains with acquired or intrinsic resistance profiles. Overall, four studies employed culture-based methods using selective media. However, none of these studies reported quantitative concentration data, such as colony-forming units per volume, limiting comparability between results and the assessment of the environmental microbial load. Considering that most currently available culture-based methods rely on selective enrichment approaches, quantitative measurements such as colony-forming units per volume are not consistently applicable or comparable across studies. The adoption of standardized reporting criteria may improve comparability between investigations and enable more robust meta-analyses, supporting the development of effective policies within the One Health framework.

In the United States, *C. auris* was detected in 7.7% of the wastewater samples analyzed using the Illumina NextSeq 2000 platform,^22^ while, through qPCR, genetic material of the species was identified in 79% of the samples,^12^ highlighting the potential of molecular detection for effective surveillance. In another study, 190 WWTPs were evaluated, with *C. auris* detected in 65 facilities by qPCR,^15^ indicating the widespread dissemination of the pathogen.

The identification of *C. auris* isolates was reported using mass spectrometry (MALDI-TOF MS).^59^ The use of colony PCR for identification was also described; however, in this case, no *C. auris* isolates were detected, and other pathogenic species such as *C. albicans*, *C. tropicalis*, and *C. krusei* were identified.^58^

Antifungal susceptibility of *C. auris* was evaluated by determining the Minimum Inhibitory Concentration (MIC). Wastewater-derived isolates demonstrated the emergence of high-level resistance to echinocandins, with anidulafungin and caspofungin MICs ≥ 32 mg/L and micafungin MICs ranging from 4 to 8 mg/L. In contrast, susceptibility to amphotericin B remained stable, with MIC values ranging from 0.25 to 0.38 mg/L.^59^ In addition, antifungal susceptibility profiling of yeasts isolated from wastewater has also been reported using the automated VITEK 2 system.^58^

The absence of validated protocols hinders the comparability of results across studies and limits the adoption of consistent laboratory practices in environmental and sanitary surveillance research. From a technical perspective, significant challenges remain regarding fungal DNA extraction from complex matrices, as well as limitations in the use of qPCR and ddPCR for the quantification and specific identification of *C. auris*. Another critical drawback is the inability of these methods to differentiate viable from nonviable cells, which prevents an accurate assessment of the actual potential for the transmission of waterborne fungal diseases. Complementary techniques, such as high-throughput genomic sequencing, may allow for greater accuracy in the characterization of fungal diversity; however, they still present high costs and require a specialized laboratory infrastructure and personnel. It was observed that in three qPCR-based studies, similar sample concentration protocols were applied, along with the use of the same sets of primers and probes, indicating a certain degree of methodological consistency among these approaches. However, qPCR cycle threshold (Ct) values were not consistently reported across studies, limiting quantitative comparisons among investigations. However, this apparent standardization should be interpreted with caution, as it is based on a still limited number of studies, which restricts the generalizability and reproducibility of these methodologies across different environmental contexts.^12,61,62^

It is important to develop studies through cultivation methods so that not only the total genetic material of *C. auris* in wastewater is known but also viability data, as these can be used in microbiological risk assessment analyses. The adoption of strategies based on selective cultivation, yeast isolation, and subsequent identification by mass spectrometry (MALDI-TOF MS) represents a promising approach to overcome some of the current limitations.^58^ The combined use of chromogenic media, antifungal supplementation, and rapid MALDI-TOF identification may significantly improve the detection of resistant *C. auris* strains in environmental samples.

### 3.5 Integrating Wastewater into One Health Surveillance: A Proposed Framework and Minimum Reporting Criteria for *C. auris*

A common challenge in the treatment of fungal infections is the emergence and spread of antifungal resistance. In this context, important advances have been observed in public health, including the strengthening of targeted surveillance, the development of faster and more accurate diagnostic tools, the introduction of new antifungal agents, and the implementation of improved antifungal stewardship practices in both clinical and agricultural settings. However, significant gaps still remain, including the need for more effective guidelines to reduce hospital- and community-acquired fungal infections, as well as the establishment of targeted early warning systems.^63^ Additionally, it remains poorly understood whether wastewater matrices and wastewater treatment plants may act as selective environments for antifungal resistance, particularly in light of the incomplete removal of certain antifungal compounds during conventional treatment processes.^64^

In this scenario, the inclusion of wastewater-based surveillance programs may represent a promising approach for early outbreak detection and for supporting preventive public health actions, contributing to the integration of environmental, human, and sanitary components within a One Health framework.

Recent genomic studies indicate that *C. auris* populations may undergo limited but ongoing genetic exchange,^65^ reinforcing the need for continuous surveillance and standardized genomic reporting within One Health frameworks. However, the lack of standardization in data generation and reporting remains a major barrier to comparability across studies. Although more than 4,000 *C. auris* genomes are currently available in public databases, only a fraction (*n* = 387) includes associated antifungal susceptibility (MIC) data, often generated using different methodologies, and many records lack essential metadata, such as geographic origin and time of isolation.^66^ These limitations hinder robust meta-analyses and the identification of consistent associations between genetic variants and antifungal resistance.

Furthermore, the integration of environmental and clinical data remains limited. However, recent evidence has strengthened the potential of wastewater surveillance as a bridge among these domains. A high-resolution, facility-level study conducted in Southern Nevada demonstrated that upstream sewage monitoring at healthcare facilities showed significantly higher sensitivity (*p* < 0.001) than sampling at wastewater treatment plants. Using amplicon sequencing and MALDI-TOF mass spectrometry, the authors identified clinically relevant resistance-associated variants in wastewater samples. In addition, whole-genome sequencing revealed >90% genomic concordance between 443 wastewater-derived genomes and 2,945 clinical isolates. Previously unreported subclades and resistance-associated mutations, including FKS1 Phe635Leu and co-occurring ERG11/FKS1 variants, were also detected in wastewater samples up to nearly five months before their identification in clinical settings. These findings highlight the potential of wastewater surveillance as a high-resolution early warning tool for outbreak detection, genomic tracking, and antifungal resistance monitoring. Although this approach has not yet been widely applied to *C. auris* or other pathogenic yeasts across different settings, these results reinforce the need for integrated surveillance strategies to better understand the transmission dynamics and resistance patterns.

In Brazil, the National Health Surveillance Agency (ANVISA) has established standardized protocols for environmental surveillance of *C. auris* in healthcare settings, including sampling of high-contact surfaces using cellulose sponge swabs, followed by selective enrichment in Sabouraud dextrose broth supplemented with 10% NaCl and antibiotics, incubation at 40 °C, and subsequent isolation on chromogenic agar media. However, these protocols were specifically developed for clinical and hospital environmental surveillance and are not directly applicable to wastewater matrices, which present substantially greater physicochemical and microbiological complexity. This highlights the current lack of standardized methodologies for the detection and monitoring of *C. auris* in wastewater samples.^67^

Therefore, based on the studies published to date, this study proposes a conceptual workflow (Figure 1) to systematize the methodological steps for the detection and monitoring of *C. auris* in wastewater. This workflow also outlines a minimum set of parameters that should be reported, including sampling conditions, enrichment and cultivation strategies, qPCR data (Ct and gc·L^–1^), isolate identification, antifungal susceptibility profiles (MIC), and associated genomic data. The adoption of standardized reporting criteria may improve comparability between investigations and enable more robust meta-analyses, supporting the development of effective policies within the One Health framework.

**Figure 1:**
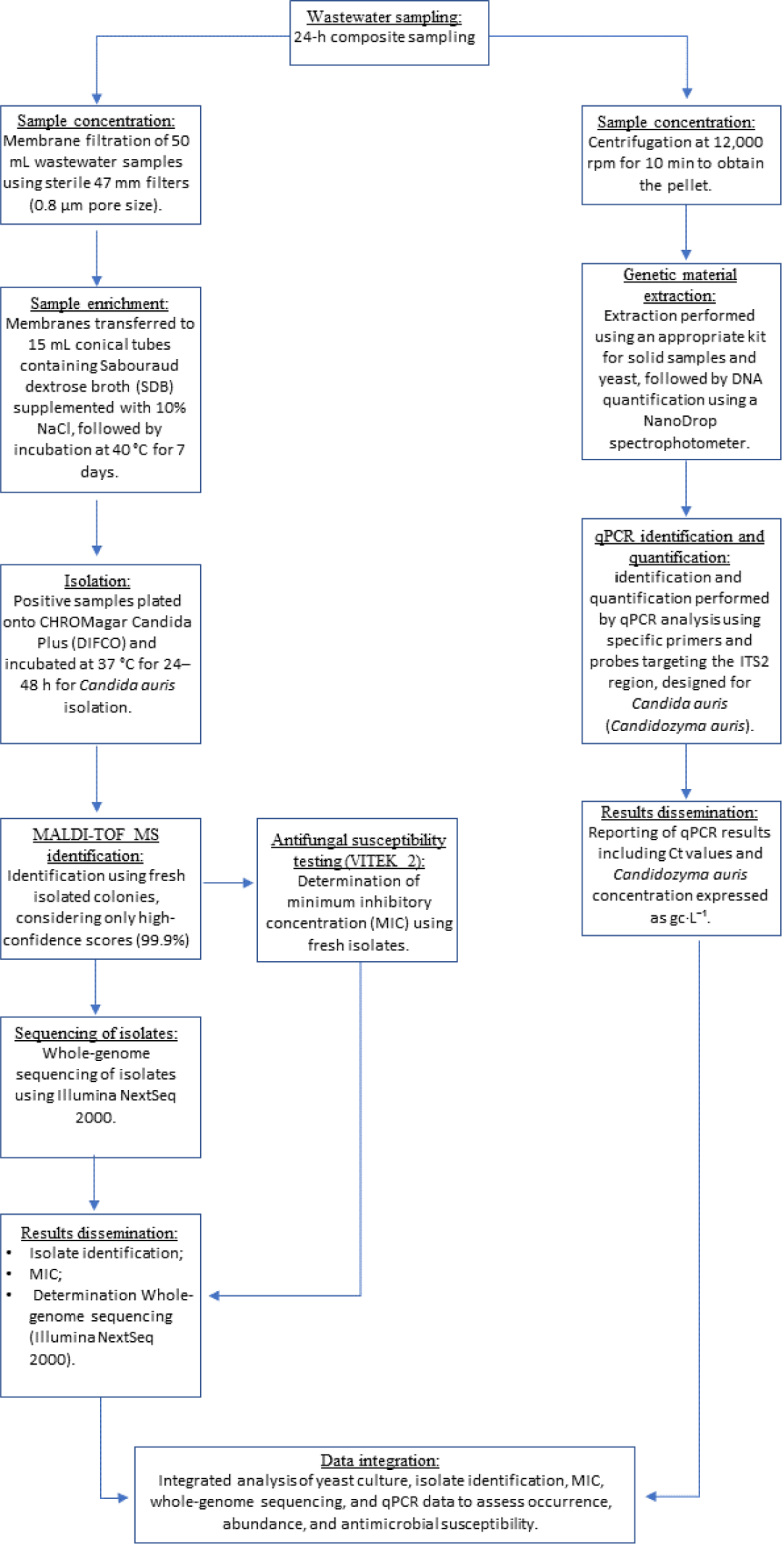
Proposed workflow for *C. auris* analysis in wastewater using culture-based methods and qPCR.

## 4 Conclusions and Future Challenges

The emergence of *C. auris* as a priority pathogen and its detection in wastewater present significant challenges for public health, the environment, and food safety. Although WWTPs play a key role in mitigating microbiological risks, the current evidence regarding the removal of *C. auris* remains limited. Available data, based on a small number of studies, are insufficient to determine the effectiveness of different treatment stages, particularly secondary processes, in eliminating viable and resistant yeasts.

The absence of specific standards for monitoring pathogenic fungi in effluents and biosolids highlights a relevant gap in current regulatory frameworks. However, the implications for environmental and occupational health remain insufficiently characterized and should be interpreted with caution. Improving the understanding of *C. auris* dynamics throughout wastewater treatment processes may support the development of safer and more sustainable management strategies aligned with the protection of human, animal, and environmental health while also informing future regulatory discussions on the inclusion of fungal pathogens in criteria for treated effluent quality and reuse practices.

Further studies are needed to develop and standardize methodologies for the detection of pathogenic yeasts in the environment. Approaches combining selective culture methods, including the use of chromogenic media and the incorporation of antifungal agents to promote the selection of resistant yeast strains, followed by isolation and identification using MALDI-TOF, may represent promising complementary strategies to data obtained through molecular techniques, such as qPCR. However, their applicability and performance in complex environmental matrices require further validation.

From a One Health perspective, the integration of surveillance strategies that consider the presence of fungi and antifungal resistance in the environment may contribute to a better understanding of *C. auris* occurrence. Potential pathways of dissemination through water, soil, the food chain, and human exposure remain to be further investigated.

Overall, this study highlights important knowledge gaps and the need for interdisciplinary efforts to better understand the environmental role of *C. auris*. The potential inclusion of this pathogen in environmental monitoring and wastewater-based epidemiology frameworks should be further explored, considering the current limitations in available data.

## References

[1] Fisher M. C., Denning D. W. (2023). The WHO Fungal Priority
Pathogens List as a Game-Changer. Nat. Rev.
Microbiol..

[2] Bongomin F., Gago S., Oladele R., Denning D. (2017). Global and
Multi-National
Prevalence of Fungal Diseases—Estimate Precision. J. Fungi.

[3] Wang X., Bing J., Zheng Q., Zhang F., Liu J., Yue H., Tao L., Du H., Wang Y., Wang H., Huang G. (2018). The First Isolate of *Candida auris* in China: Clinical
and Biological Aspects. Emerg. Microbes Infect..

[4] World Health Organization WHO Fungal Priority Pathogens List to Guide Research, Development and Public Health Action; World Health Organization, 2022.

[5] Lockhart S. R., Etienne K. A., Vallabhaneni S., Farooqi J., Chowdhary A., Govender N. P., Colombo A. L., Calvo B., Cuomo C. A., Desjardins C. A., Berkow E. L., Castanheira M., Magobo R. E., Jabeen K., Asghar R. J., Meis J. F., Jackson B., Chiller T., Litvintseva A. P. (2017). Simultaneous
Emergence of Multidrug-Resistant *Candida auris* on
3 Continents Confirmed by Whole-Genome Sequencing and Epidemiological
Analyses. Clin. Infect. Dis..

[6] Suphavilai C., Ko K. K. K., Lim K. M., Tan M. G., Boonsimma P., Chu J. J. K., Goh S. S., Rajandran P., Lee L. C., Tan K. Y., Shaik
Ismail B. B., Aung M. K., Yang Y., Sim J. X. Y., Venkatachalam I., Cherng B. P. Z., Spruijtenburg B., Chan K. S., Oon L. L. E., Tan A. L., Tan Y. E., Wijaya L., Tan B. H., Ling M. L., Koh T. H., Meis J. F., Tsui C. K. M., Nagarajan N. (2024). Detection
and Characterisation of a Sixth *Candida
auris* Clade in Singapore: A Genomic and Phenotypic Study. Lancet Microbe.

[7] Fan S., Yue H., Zheng Q., Bing J., Tian S., Chen J., Ennis C. L., Nobile C. J., Huang G., Du H. (2021). Filamentous
Growth Is a General Feature of *Candida auris* Clinical
Isolates. Med. Mycol..

[8] Ferrara G. A., Spruijtenburg B., Meijer E. F. J., Meis J. F., Ceballos-Garzon A., Caceres D. H. (2025). First Report of *Candida (Candidozyma) auris* Infections in a Human and a Dolphin from the Dominican Republic:
A One Health Perspective. Med. Mycol. Case Rep..

[9] Grassi A., Rigamonti S., Danesi P., Olivieri E., Sgubin S., Prati P. (2025). First Report
of *Candidozyma auris* (*Candida
auris*) in a Cat: A Case of Persistent Shedding. Med. Mycol. Case Rep..

[10] Yadav A., Wang Y., Jain K., Panwar V. A. R., Kaur H., Kasana V., Xu J., Chowdhary A. (2023). *Candida
auris* in Dog Ears. J. Fungi.

[11] White T. C., Esquivel B. D., Rouse
Salcido E. M., Schweiker A. M., dos Santos A. R., Gade L., Petro E., KuKanich B., KuKanich K. S. (2024). *Candida auris* Detected in the Oral
Cavity of a Dog in Kansas. mBio.

[12] Barber C., Crank K., Papp K., Innes G. K., Schmitz B. W., Chavez J., Rossi A., Gerrity D. (2023). Community-Scale
Wastewater
Surveillance of *Candida auris* during an Ongoing Outbreak
in Southern Nevada. Environ. Sci. Technol..

[13] Satoh K., Makimura K., Hasumi Y., Nishiyama Y., Uchida K., Yamaguchi H. (2009). *Candida auris* Sp.
Nov., a Novel Ascomycetous Yeast Isolated from the External Ear Canal
of an Inpatient in a Japanese Hospital. Microbiol.
Immunol..

[14] Arora P., Singh P., Wang Y., Yadav A., Pawar K., Singh A., Padmavati G., Xu J., Chowdhary A. (2021). Environmental
Isolation of *Candida auris* from the Coastal Wetlands
of Andaman Islands, India. mBio.

[15] Zulli A., Chan E. M. G., Shelden B., Duong D., Xu X.-R. S., White B. J., Wolfe M. K., Boehm A. B. (2024). Prospective
Study
of *Candida auris* Nucleic Acids in Wastewater Solids
in 190 Wastewater Treatment Plants in the United States Suggests Widespread
Occurrence. mBio.

[16] Benedict K., Forsberg K., Gold J. A. W., Baggs J., Lyman M. (2023). *Candida
auris* – Associated Hospitalizations, United States,
2017–2022. Emerg. Infect. Dis..

[17] Bravo
Ruiz G., Ross Z. K., Gow N. A. R., Lorenz A. (2020). Pseudohyphal Growth
of the Emerging Pathogen *Candida auris* Is Triggered
by Genotoxic Stress through the S Phase Checkpoint. mSphere.

[18] Day A. M., McNiff M. M., da Silva
Dantas A., Gow N. A. R., Quinn J. (2018). Hog1 Regulates
Stress Tolerance and Virulence in the Emerging Fungal Pathogen *Candida auris*. mSphere.

[19] Stephenson J. C., Garza D. R., Bouklas T. (2023). A Fungus for
Our Time: *Candida
auris* Emerges into the Anthropocene. Curr. Trop. Med. Rep..

[20] Jennifer Z.-A., Sonia A.-D., Francesca E.-B., Nadia F.-M., Suanny M.-R. (2024). Advanced
Oxidation Processes by UV/H2O2 for the Removal of Anionic Surfactants
in a Decentralized Wastewater Treatment Plant in Ecuador. Water Sci. Technol..

[21] Casalini G., Giacomelli A., Antinori S. (2024). The WHO Fungal Priority Pathogens
List: A Crucial Reappraisal to Review the Prioritisation. Lancet Microbe.

[22] Rossi A., Chavez J., Iverson T., Hergert J., Oakeson K., LaCross N., Njoku C., Gorzalski A., Gerrity D. (2023). *Candida auris* Discovery
through Community
Wastewater Surveillance during Healthcare Outbreak, Nevada, USA, 2022. Emerg. Infect. Dis..

[23] Ni B.-J., Yan X., Dai X., Liu Z., Wei W., Wu S.-L., Xu Q., Sun J. (2020). Ferrate Effectively Removes Antibiotic Resistance Genes
from Wastewater through Combined Effect of Microbial DNA Damage and
Coagulation. Water Res..

[24] Zumla A., Azhar E. I., Hui D. S., Shafi S., Petersen E., Memish Z. A. (2018). Global Spread of Antibiotic-Resistant
Bacteria and
Mass-Gathering Religious Events. Lancet Infect.
Dis..

[25] Chatterjee A., Modarai M., Naylor N. R., Boyd S. E., Atun R., Barlow J., Holmes A. H., Johnson A., Robotham J. V. (2018). Quantifying
Drivers of Antibiotic Resistance in Humans: A Systematic Review. Lancet Infect. Dis..

[26] Queenan K., Häsler B., Rushton J. (2016). A One Health Approach
to Antimicrobial
Resistance Surveillance: Is There a Business Case for It?. Int. J. Antimicrob. Agents.

[27] Sims N., Kasprzyk-Hordern B. (2020). Future Perspectives
of Wastewater-Based Epidemiology:
Monitoring Infectious Disease Spread and Resistance to the Community
Level. Environ. Int..

[28] Larsson D. G. J., Flach C.-F. (2022). Antibiotic Resistance in the Environment. Nat. Rev. Microbiol..

[29] Hernando-Amado S., Coque T. M., Baquero F., Martínez J. L. (2020). Antibiotic
Resistance: Moving From Individual Health Norms to Social Norms in
One Health and Global Health. Front. Microbiol..

[30] Karkman A., Do T. T., Walsh F., Virta M. P. J. (2018). Antibiotic-Resistance
Genes in Waste Water. Trends Microbiol..

[31] Gholizadeh A., Khiadani M., Foroughi M., Alizade Siuki H., Mehrfar H. (2023). Wastewater Treatment Plants: The Missing Link in Global
One-Health Surveillance and Management of Antibiotic Resistance. J. Infect. Public Health.

[32] Assress H. A., Selvarajan R., Nyoni H., Ogola H. J. O., Mamba B. B., Msagati T. A. M. (2021). Azole
Antifungal Resistance in Fungal Isolates from
Wastewater Treatment Plant Effluents. Environ.
Sci. Pollut. Res..

[33] Andrade E. F., Poester V. R., Esperon B. M., Trápaga M. R., Hidalgo J. E. D., Ferreira F. B., de Souza M. M., Severo C. B., Groll A. V., Xavier M. O. (2025). Pathogenic *Aspergillus* Spp. and *Candida* Spp. in Coastal
Waters from Southern
Brazil: An One Health Approach. Braz. J. Microbiol..

[34] Brandão J., Gangneux J. P., Arikan-Akdagli S., Barac A., Bostanaru A. C., Brito S., Bull M., Çerikçioğlu N., Chapman B., Efstratiou M. A., Ergin Ç., Frenkel M., Gitto A., Gonçalves C. I., Guégan H., Gunde-Cimerman N., Güran M., Irinyi L., Jonikaitė E., Kataržytė M., Klingspor L., Mares M., Meijer W. G., Melchers W. J. G., Meletiadis J., Meyer W., Nastasa V., Babič M. N., Ogunc D., Ozhak B., Prigitano A., Ranque S., Rusu R. O., Sabino R., Sampaio A., Silva S., Stephens J. H., Tehupeiory-Kooreman M., Tortorano A. M., Velegraki A., Veríssimo C., Wunderlich G. C., Segal E. (2021). Mycosands: Fungal Diversity and Abundance
in Beach Sand and Recreational Waters – Relevance to Human
Health. Sci. Total Environ..

[35] Marathe N. P., Pal C., Gaikwad S. S., Jonsson V., Kristiansson E., Larsson D. G. J. (2017). Untreated Urban
Waste Contaminates Indian River Sediments
with Resistance Genes to Last Resort Antibiotics. Water Res..

[36] Lekunberri I., Balcázar J. L., Borrego C. M. (2018). Metagenomic Exploration Reveals a
Marked Change in the River Resistome and Mobilome after Treated Wastewater
Discharges. Environ. Pollut..

[37] Wang J.-Y., An X.-L., Huang F.-Y., Su J.-Q. (2020). Antibiotic Resistome
in a Landfill Leachate Treatment Plant and Effluent-Receiving River. Chemosphere.

[38] Zayed M., Soliman M., Kalil R., Saad T. (2016). Isolation of *Candida albicans* from naturally infected freshwater fish. Kafrelsheikh Vet. Med. J..

[39] Yanong R. P. E. (2003). Fungal
Diseases of Fish. Vet. Clin. North Am.: Exot.
Anim. Pract..

[40] Gazal L. E., de Brito K. C. T., Kobayashi R., Nakazato G., Cavalli L., Otutumi L. K., de Brito B. G. (2020). Antimicrobials
and Resistant Bacteria
in Global Fish Farming and the Possible Risk for Public Health. Arq. Inst. Biol. (Sao. Paulo).

[41] Møller S. A., Frederiksen M. W., Rasmussen P. U., Østergaard S. K., Nielsen J. L., Madsen A. M. (2025). Characterization
of Bioaerosol Exposures
in Wastewater Treatment Plant Workers and Serum Levels of Lung and
Inflammatory Markers. J. Hazard. Mater..

[42] He P., Wei S., Shao L., Lü F. (2019). Aerosolization Behavior of Prokaryotes
and Fungi during Composting of Vegetable Waste. Waste Manag..

[43] CONAMA Resolução No. 430, de 13 de Maio de 2011: dispõe Sobre as Condições e Padrões de Lançamento de Efluentes, Complementa e Altera a Resolução No. 357/2005 Do Conselho Nacional Do Meio Ambiente; Ministério do Meio Ambiente, 2011.

[44] European Parliament Regulation (EU) 2020/741 of the European Parliament and of the Council of 25May 2020on Minimum Requirements for Water Reuse; European Union, 2020.

[45] U.S. Environmental Protection Agency. Guidelines for Water Reuse EPA/600/R-12/618; National Service Center for Environmental Publications 2012.

[46] Finley R. L., Collignon P., Larsson D. G. J., McEwen S. A., Li X.-Z., Gaze W. H., Reid-Smith R., Timinouni M., Graham D. W., Topp E. (2013). The Scourge
of Antibiotic Resistance:
The Important Role of the Environment. Clin.
Infect. Dis..

[47] Forsberg K. J., Reyes A., Wang B., Selleck E. M., Sommer M. O. A., Dantas G. (2012). The Shared Antibiotic Resistome of Soil Bacteria and
Human Pathogens. Science(1979).

[48] U.S. Environmental Protection Agency. Standards for the Use or Disposal of Sewage Sludge (40 CFR Part 503). U.S. Government Printing Office 1993.

[49] European Economic Community Council Directive 86/278/EEC of 12 June 1986 on the Protection of the Environment, and in Particular of the Soil, When Sewage Sludge Is Used in Agriculture; Official Journal of the European Communities, 1986; pp 6–12.

[50] World Health Organization Guidelines for the Safe Use of Wastewater, Excreta, and Greywater; World Health Organization, 2006.

[51] Conselho Nacional do Meio Ambiente Resolução No. 498, de 19 de Agosto de 2020: define Critérios e Procedimentos Para Produção e Aplicação de Biossólidos Em Solos, e Dá Outras Providências; Conselho Nacional do Meio Ambiente, 2020.

[52] Secretário de Estado da Agricultura Instrução Normativa - SEAPI N^o^ 07/2016; Estado do Rio Grande do Sul Secretaria da Agricultura, Pecuária e Irrigação Departamento de Defesa Agropecuária, 2016.

[53] Pruden A. (2014). Balancing
Water Sustainability and Public Health Goals in the Face of Growing
Concerns about Antibiotic Resistance. Environ.
Sci. Technol..

[54] Manaia C. M., Rocha J., Scaccia N., Marano R., Radu E., Biancullo F., Cerqueira F., Fortunato G., Iakovides I. C., Zammit I., Kampouris I., Vaz-Moreira I., Nunes O. C. (2018). Antibiotic Resistance in Wastewater
Treatment Plants: Tackling the Black Box. Environ.
Int..

[55] Cadnum J. L., Shaikh A. A., Piedrahita C. T., Jencson A. L., Larkin E. L., Ghannoum M. A., Donskey C. J. (2018). Relative Resistance of the Emerging
Fungal Pathogen *Candida auris* and Other *Candida* Species to Killing by Ultraviolet Light. Infect.
Control Hosp. Epidemiol..

[56] de
Groot T., Chowdhary A., Meis J. F., Voss A. (2019). Killing of *Candida auris* by UV-C: Importance of Exposure Time and Distance. Mycoses.

[57] Ponnachan P., Vinod V., Pullanhi U., Varma P., Singh S., Biswas R., Kumar A. (2019). Antifungal Activity of Octenidine
Dihydrochloride and Ultraviolet-C Light against Multidrug-Resistant *Candida auris*. J. Hosp. Infect..

[58] Corrêa-Moreira D., da Costa G. L., de Lima Neto R. G., Pinto T., Salomão B., Fumian T. M., Mannarino C. F., Prado T., Miagostovich M. P., de Souza Ramos L., Souza dos Santos A. L., Oliveira M. M. E. (2024). Screening of *Candida* Spp. in Wastewater in Brazil during COVID-19 Pandemic:
Workflow for Monitoring Fungal Pathogens. BMC
Biotechnol..

[59] Chang, C.-L. ; Moshi, M. A. ; Nguyen, Q.-H. ; Oh, J. ; Nguyen, H. ; Paranjape, P. ; Abushanab, M. ; Tang, A. J. ; Itorralba, J. Y. ; Massic, L. ; Khan, E. ; Lockett, C. ; Kan, H.-Y. ; Pandori, M. ; Gerrity, D. ; Vo, V. ; Nguyen, T. ; Hess, D. ; Oh, E. C. Wastewater Intelligence Predicts the Emergence of Clinically-Relevant and Drug-Resistant Candidozyma auris at Healthcare Facilities. Nat. Commun. 2026, 10.1038/s41467-026-71960-5. PMC13280361 42000719

[60] Babler K., Sharkey M., Arenas S., Amirali A., Beaver C., Comerford S., Goodman K., Grills G., Holung M., Kobetz E., Laine J., Lamar W., Mason C., Pronty D., Reding B., Schürer S., Schaefer Solle N., Stevenson M., Vidović D., Solo-Gabriele H., Shukla B. (2023). Detection of the Clinically Persistent,
Pathogenic Yeast Spp. *Candida auris* from Hospital
and Municipal Wastewater in Miami-Dade County, Florida. Sci. Total Environ..

[61] Nwaubani D. A., Baral R., Solomon T., Idris O., Sherchan S. P. (2025). Wastewater
Surveillance of *Candida auris* in Baltimore. Int. J. Hyg. Environ. Health.

[62] Chavez J., Crank K., Barber C., Gerrity D., Iverson T., Mongillo J., Weil A., Rider L., Lacross N., Oakeson K., Rossi A. (2024). Early Introductions
of *Candida
auris* Detected by Wastewater Surveillance, Utah, USA, 2022–2023. Emerg. Infect. Dis..

[63] Wang Y., Han L., Gong J., Liu L., Miao B., Xu J. (2025). Research Advances
and Public Health Strategies in China on WHO Priority Fungal Pathogens. Mycology.

[64] Sabino R., Antunes F., Araujo R., Bezerra A. R., Brandão J., Carneiro C., Carvalho A., Carvalho D., Conceição I. C., Cota
Medeiros F., Cruz C., Duarte E., Holum S., Matos O., Maltez F., Mendonça A., Moura G., Pereira A., Fortuna Rodrigues C., Teixeira P., Valdoleiros S. R., Veríssimo C., Viegas C. (2025). Addressing Critical Fungal Pathogens Under a One Health
Perspective: Key Insights from the Portuguese Association of Medical
Mycology. Mycopathologia.

[65] Wang Y., Xu J. (2022). Population Genomic
Analyses Reveal Evidence for Limited Recombination
in the Superbug *Candida auris* in Nature. Comput. Struct. Biotechnol. J..

[66] Wang Y., Xu J. (2024). Associations between
Genomic Variants and Antifungal Susceptibilities
in the Archived Global *Candida auris* Population. J. Fungi.

[67] Brazilian Health Regulatory Agency (ANVISA). Technical Note GVIMS/GGTES/ANVISA No. 02/2022: guidelines for Identification, Prevention and Control of Candida auris Infections in Healthcare Services; Brazilian Health Regulatory Agency (ANVISA), 2024.

